# Epidemiologic Characteristics of 1.4 Million Multiplex PCR Tests for 12 Urogenital and Sexually Transmitted Infection Pathogens in Korea (2021–2024)

**DOI:** 10.3390/pathogens14111073

**Published:** 2025-10-22

**Authors:** Soyoun Shin

**Affiliations:** Daejeon & Chungcheong Reference Lab, Seegene Medical Foundation, Daejeon 35203, Republic of Korea; drshin@mf.seegene.com

**Keywords:** sexually transmitted infections (STIs), urogenital flora, epidemiology, multiplex PCR, nucleic acid amplification test (NAAT), nationwide surveillance, co-infection, prevalence, Korea

## Abstract

Sexually transmitted infections (STIs) remain a global health concern, but large-scale multiplex PCR surveillance data are limited. This study aimed to characterize sex- and age-specific distributions, temporal patterns, and co-infection dynamics of 12 urogenital and sexually transmitted infection (STI) pathogens in Korea. We retrospectively analyzed 1,399,431 multiplex PCR test records (902,713 females, 496,718 males) collected nationwide between 2021 and 2024. Positivity rates were stratified by sex, age, month, season, and year. Co-infection coverage and inter-pathogen correlations were assessed; *φ* coefficients ≥0.20 were considered relevant. Overall, 67.23% of tests were positive for at least one pathogen. Annually, positivity rates for most pathogens, including the six traditional STIs (*N. gonorrhoeae*, *C. trachomatis*, *M. genitalium*, *T. vaginalis*, *T. pallidum*, and HSV II), showed a significant decline over the four-year period (*p* < 0.0001). Females had higher positivity than males (77.73% vs. 48.14%, *p* < 0.0001), largely driven by *G. vaginalis* (64.70%), *U. parvum* (41.37%), and *C. albicans* (18.07%), whereas traditional STIs, except *T. vaginalis* and HSV II, were more frequent in males (*p* < 0.0001). Adolescents and young adults carried the highest burden of traditional STIs such as *C. trachomatis* and *N. gonorrhoeae* (*p* < 0.0001). In females, *C. albicans* decreased with age (32.17% in 10s to 6.45% in 80s) but increased annually (*p* = 0.0058), while HSV II positivity significantly declined in males over time (*p* = 0.0038). No seasonal variation was observed (*p* > 0.90). Co-infections were predominantly commensal-driven, with *U. parvum*/*G. vaginalis* being the most frequent pair in females, and *U. urealyticum*/*G. vaginalis* being frequent in males (8.24% in 10s, 10.48% in 20s). Traditional STI co-infections were rare but concentrated in adolescents, with *C. trachomatis*/*N. gonorrhoeae* reaching 4.25% in males. Correlation analysis confirmed strong associations among commensals (*φ* = 0.24–0.35) and moderate correlations involving *C. trachomatis* in youth (*φ* = 0.25–0.28), with *G. vaginalis* consistently identified as the central hub organism across all age groups. This nationwide four-year analysis highlights declining prevalence of traditional STIs, sex-specific STI patterns, distinct age-specific distributions, and commensal-driven co-infection patterns. The findings underscore the need for age- and sex-tailored screening strategies, particularly in adolescents, young adults, and women of reproductive age, and highlight the value of multiplex PCR for STI surveillance.

## 1. Introduction

Sexually transmitted infections (STIs) remain a major global public health issue, with far-reaching implications for reproductive health, maternal and neonatal outcomes, and overall infectious disease burden. According to the World Health Organization (WHO), more than 1 million curable STIs are acquired daily, with *Chlamydia trachomatis*, *Neisseria gonorrhoeae*, *Treponema pallidum*, and *Trichomonas vaginalis* among the most commonly reported sexually transmitted pathogens worldwide [1]. These pathogens contribute not only to symptomatic infections but also to chronic reproductive tract conditions, infertility, and vertical transmission risks. Co-infections involving Mycoplasma and Ureaplasma species are increasingly recognized for their potential to influence disease severity and treatment outcomes [2].

In Korea, the incidence of STIs has fluctuated over time, influenced by changes in sexual behavior, accessibility of diagnostic services, and the implementation of public health interventions. The Korea Disease Control and Prevention Agency (KDCA) has reported temporal declining trends in bacterial STIs such as *C. trachomatis* and *N. gonorrhoeae*, while viral STIs including herpes simplex virus and human papillomavirus have continued to rise during 2019–2020 under the COVID-19 pandemic crisis, suggesting further evaluation [3,4].

Recent advances in molecular diagnostics, particularly multiplex polymerase chain reaction (PCR), have enabled simultaneous detection of multiple STI pathogens with high sensitivity and specificity. The CDC and WHO recommend nucleic acid amplification tests (NAATs) as the gold standard for STI diagnosis, especially in asymptomatic individuals [5]. In Korea, multiplex PCR testing is widely utilized at centralized reference laboratories such as the Seegene Medical Foundation, which receives nationwide clinical testing referrals.

This study aims to provide a comprehensive analysis of 12 urogenital and sexually transmitted infection (STI) pathogens over a four-year period (2021–2024) in Korea, leveraging large-scale multiplex PCR data. We examine positivity rates by sex, age, month, season, and year, and explore co-infection patterns to better understand the characteristics of urogenital and STI pathogens in the population.

## 2. Materials and Methods

### 2.1. Study Design and Data Sources

This retrospective cross-sectional study analyzed the PCR test results for 12 urogenital and STI pathogens, collected between January 2021 and December 2024 by the Seegene Medical Foundation.

The data represents nationwide clinical testing request submitted by various primary, secondary, and tertiary medical institutions across Korea. Specimen types included urine samples for male patients and cervicovaginal swabs for female patients. All tests were ordered as part of routine clinical care and all data were anonymized prior to analysis. DNA extraction was performed using the MagNA Pure 96 System (Roche Diagnostics, Mannheim, Germany), and amplification was conducted with the Anyplex™ II STI-12 Detection assay (Seegene, Seoul, Korea) and the CFX96 Real-Time PCR System (Bio-Rad Laboratories, Inc., Irvine, CA, USA) according to the manufacturer’s instructions.

This study was conducted in accordance with the Declaration of Helsinki and approved by the Institutional Review Board of Seegene Medical Foundation (SMF-IRB-2024-001, approved on 8 February 2024; SMF-IRB-2025-007, approved on 8 May 2025).

### 2.2. Pathogens and Diagnostic Method

Multiplex real-time PCR testing targeted 12 urogenital and STI pathogens: *Neisseria gonorrhoeae*, *Chlamydia trachomatis*, *Ureaplasma urealyticum*, *Mycoplasma genitalium*, *Mycoplasma hominis*, *Trichomonas vaginalis*, *Ureaplasma parvum*, *Gardnerella vaginalis*, *Treponema pallidum*, *Candida albicans*, Herpes simplex virus type I, and Herpes simplex virus type II. The Anyplex™ II STI-12 Detection kit (Seegene, Seoul, Korea) was used, following manufacturer’s protocol and the standard operation procedure of the Seegene Medical Foundation.

### 2.3. Variables and Statistical Analysis

Positivity rates were analyzed by sex, 10-year age intervals, month, season (spring, summer, fall, winter), year, and co-infection patterns. The following statistical methods were applied: chi-square tests for categorical comparisons, independent t-tests, one-way ANOVA, and simple linear regression for trend analysis.

### 2.4. Correlation Analysis of Co-Infections

Pairwise associations between pathogens were evaluated using the *φ* (phi) coefficient, which is mathematically equivalent to the Pearson correlation coefficient for binary variables (presence or absence of each pathogen).

For each pair of pathogens within each age–sex stratum, a 2 × 2 contingency table was constructed, and *φ* was calculated using the formula:

*φ* = (ad − bc)/√[(a + b)(c + d)(a + c)(b + d)]

where a = both pathogens positive, b = pathogen A positive only, c = pathogen B positive only, and d = both negative.

Values of *φ* range from –1 to +1, with positive values indicating a tendency for co-occurrence and negative values indicating mutual exclusivity. The strength of association was interpreted according to Cohen’s criteria: |*φ*| < 0.10 = negligible, 0.10–0.19 = weak, 0.20–0.29 = moderate, and ≥0.30 = strong correlation.

To reduce spurious associations, only *φ* values ≥ 0.20 were considered clinically meaningful and summarized in the main text.

## 3. Results

### 3.1. Yearly Trends (Table 1)

A total of 1,399,431 PCR test records were analyzed. Annually, the total number of 12 urogenital and STI pathogen tests increased from 311,478 in 2021, 355,881 in 2022, 378,051 in 2023 to 374,021 in 2024. Yearly changes in test volume showed no statistically significant difference between females and males over the study period (*p* = 0.3096, *t*-test).

The overall positivity rate for detection of at least one pathogen among the 12 tested targets and the six traditional STIs was 67.23% and 10.54%. Significant year-to-year decrease in positivity rates were observed for most pathogens, with a general trend of decrease including *N. gonorrhoeae*, *C. trachomatis*, *U. urealyticum*, *M. genitalium*, *M. hominis*, *T. vaginalis*, *U. parvum*, and *G. vaginalis* in both sexes (*p* < 0.0001). Notably, positivity for *C. albicans* significantly increased in females (*p* = 0.0058), while HSV II positivity decreased in males over time (*p* = 0.0038), as confirmed by linear regression analysis.

**Table 1:** Positivity rates of the 12 urogenital and STI pathogens by sex and year (n., %).

Sex	Year	Test n.	Overall	6 STIs *	NG	CT	UU	MG	MH	TV	UP	GV	TP	CA	HSV I	HSV II
Female	2021	209,009	168,428	22,510	837	7983	36,136	6131	24,817	1443	93,418	142,942	46	35,218	863	8853
			80.58	10.77	0.40	3.82	17.29	2.93	11.87	0.69	44.70	68.39	0.02	16.85	0.41	4.24
	2022	217,831	172,207	21,333	603	7103	36,537	5704	24,421	1302	93,499	144,888	53	38,313	1008	8865
			79.06	9.79	0.28	3.26	16.77	2.62	11.21	0.60	42.92	66.51	0.02	17.59	0.46	4.07
	2023	241,364	185,530	21,608	580	6323	38,828	5928	24,077	1211	97,529	152,830	36	44,827	1039	9678
			76.87	8.95	0.24	2.62	16.09	2.46	9.98	0.50	40.41	63.32	0.01	18.57	0.43	4.01
	2024	234,509	175,526	20,381	491	5680	35,498	5219	21,279	1022	88,964	143,405	22	44,794	1000	9781
			74.85	8.69	0.21	2.42	15.14	2.23	9.07	0.44	37.94	61.15	0.01	19.10	0.43	4.17
	Sub- total	902,713	701,691	85,832	2511	27,089	146,999	22,982	94,594	4978	373,410	584,065	157	163,152	3910	37,177
			77.73	9.51	0.28	3.00	16.28	2.55	10.48	0.55	41.37	64.70	0.02	18.07	0.43	4.12
Male	2021	102,469	54,049	15,262	3133	8404	16,474	3986	6279	244	15,829	33,690	65	773	287	1473
			52.75	14.89	3.06	8.20	16.08	3.89	6.13	0.24	15.45	32.88	0.06	0.75	0.28	1.44
	2022	118,050	58,227	15,129	2579	8312	17,622	4066	6327	219	16,899	36,441	82	914	402	1622
			49.32	12.82	2.18	7.04	14.93	3.44	5.36	0.19	14.32	30.87	0.07	0.77	0.34	1.37
	2023	136,687	64,153	16,022	2590	8564	19,417	4535	6915	247	18,291	39,531	65	1028	421	1743
			46.93	11.72	1.89	6.27	14.21	3.32	5.06	0.18	13.38	28.92	0.05	0.75	0.31	1.28
	2024	139,512	62,694	15,282	2434	8122	18,489	4371	6514	208	17,777	38,620	60	1290	447	1766
			44.94	10.95	1.74	5.82	13.25	3.13	4.67	0.15	12.74	27.68	0.04	0.92	0.32	1.27
	Sub- total	496,718	239,123	61,695	10,736	33,402	72,002	16,958	26,035	918	68,796	148,282	272	4005	1557	6604
			48.14	12.42	2.16	6.72	14.50	3.41	5.24	0.18	13.85	29.85	0.05	0.81	0.31	1.33
Total		1,399,431	940,814	147,527	13,247	60,491	219,001	39,940	120,629	5896	442,206	732,347	429	167,157	5467	43,781
			67.23	10.54	0.95	4.32	15.65	2.85	8.62	0.42	31.60	52.33	0.03	11.94	0.39	3.13

* 6 STIs; *N. gonorrhoeae*, *C. trachomatis*, *M. genitalium*, *T. vaginalis*, *T. pallidum*, HSV II. Abbreviations: NG, *Neisseria gonorrhoeae*; CT, *Chlamydia trachomatis*; UU, *Ureaplasma urealyticum*; MG, *Mycoplasma genitalium*; MH, *Mycoplasma hominis*; TV, *Trichomonas vaginalis*; UP, *Ureaplasma parvum*; GV, *Gardnerella vaginalis*; TP, *Treponema pallidum*; CA, *Candida albicans*; HSVI, Herpes simplex virus type I; HSVII, Herpes simplex virus type II.

### 3.2. Monthly and Seasonal Trends

No statistically significant differences were observed by month (*p* > 0.95) or season by Korean 4-season classification (*p* > 0.90) for any pathogen in either sex, indicating absence of seasonal variation in 12 urogenital and STI pathogen positivity.

### 3.3. Sex-Based Comparison (Table 1)

Of the 1,399,431 tests, 902,713 (64.5%) were from females and 496,718 (35.5%) from males. The median age was 39.2 years (IQR: 29.8–53.7) in males, and 39.3 years (IQR: 29.5–50.7) in females, with no statistically significant difference.

Females showed higher positivity for at least one pathogen (77.73% vs. 48.14%, *p* < 0.0001), whereas detection of the six traditional STIs was higher in males (12.42% vs. 9.51%, *p* < 0.0001). Consistently, across the four-year period (2021–2024), significant sex-specific differences were observed for all 12 urogenital and STI pathogens (*p* < 0.0001). Females had higher positivity for urogenital flora-associated organisms, notably *G. vaginalis* (64.70% vs. 29.85%), *U. parvum* (41.37% vs. 13.85%), and *C. albicans* (18.07% vs. 0.81%), as well as *U. urealyticum*, *M. hominis*, *T. vaginalis*, HSV I, and HSV II (all *p* < 0.0001). In contrast, males demonstrated higher positivity for traditional STIs including *N. gonorrhoeae* (2.16% vs. 0.28%), *C. trachomatis* (6.72% vs. 3.00%), *M. genitalium* (3.41% vs. 2.55%), and *T. pallidum* (0.05% vs. 0.02%) (all *p* < 0.0001).

### 3.4. Age Group Trends in Urogential and STI Positivity (Table 2)

Age-stratified analysis confirmed significant differences in positivity across age groups for the majority of pathogens in both sexes (all *p* < 0.05 by ANOVA).

Adolescents (10s) had the highest positivity for *C. trachomatis*, *N. gonorrhoeae*, and *U. urealyticum*, with rates declining markedly in older age groups (all *p* < 0.0001). *M. hominis* and *M. genitalium* also showed significant age-dependent trends, peaking in the 10s among females and the 20s among males.

*C. albicans* showed a marked age-dependent decrease in females, from 32.17% in the 10s to 6.45% in the 80s. Viral STIs (HSV I and HSV II) did not show age-specific patterns, with relatively stable positivity across age groups.

**Table 2:** Age-specific positivity rates of the 12 urogenital and STI pathogens by sex (n, %).

Sex	Age	Test n.	Overall	6 STIs *	NG	CT	UU	MG	MH	TV	UP	GV	TP	CA	HSV I	HSV II
Female	10s	18,848	16,816	4664	427	2877	5310	1719	4275	231	9124	14,832	38	6064	210	761
			89.22	24.75	2.27	15.26	28.17	9.12	22.68	1.23	48.41	78.69	0.20	32.17	1.11	4.04
	20s	208,330	182,216	33,504	1224	15,277	45,990	12,156	29,590	1073	103,435	155,074	85	56,518	1450	8908
			87.47	16.08	0.59	7.33	22.08	5.83	14.20	0.52	49.65	74.44	0.04	27.13	0.70	4.28
	30s	231,595	184,972	17,705	426	4817	35,414	5332	20,024	939	100,708	149,171	15	46,734	931	7517
			79.87	7.64	0.18	2.08	15.29	2.30	8.65	0.41	43.48	64.41	0.01	20.18	0.40	3.25
	40s	196,196	158,126	11,906	210	2132	30,041	2675	19,973	1204	91,550	130,503	7	34,902	624	6265
			80.60	6.07	0.11	1.09	15.31	1.36	10.18	0.61	46.66	66.52	0.00	17.79	0.32	3.19
	50s	145,292	105,886	10,062	148	1325	21,318	928	15,105	1029	51,914	91,135	10	13,067	459	6971
			72.88	6.93	0.10	0.91	14.67	0.64	10.40	0.71	35.73	62.73	0.01	8.99	0.32	4.80
	60s	72,189	40,341	5795	66	528	7200	150	4593	419	13,679	33,206	2	4100	166	4782
			55.88	8.03	0.09	0.73	9.97	0.21	6.36	0.58	18.95	46.00	0.00	5.68	0.23	6.62
	70s	22,903	10,455	1737	9	115	1429	19	851	70	2431	8087	0	1292	45	1546
			45.65	7.58	1.00	0.50	6.24	0.08	3.72	0.31	10.61	35.31	0.00	5.64	0.20	6.75
	80s	7360	2879	459	1	18	297	3	183	13	569	2057	0	475	25	427
			39.12	6.24	0.01	0.24	4.04	0.04	2.49	0.18	7.73	27.95	0.00	6.45	0.34	5.80
	Sub-total	902,713	701,691	85,832	2511	27,089	146,999	22,982	94,594	4978	373,410	584,065	157	163,152	3910	37,177
			77.73	9.51	0.28	3.00	16.28	2.55	10.48	0.55	41.37	64.70	0.02	18.07	0.43	4.12
Male	10s	6590	3552	2038	609	1394	1280	406	421	11	784	1615	8	74	55	126
			53.90	30.93	9.24	21.15	19.42	6.16	6.39	0.17	11.90	24.51	0.12	1.12	0.83	1.91
	20s	113,175	65,692	26,556	4514	16,047	20,487	7196	7385	118	16,576	35,679	112	1045	648	2327
			58.04	23.46	3.99	14.18	18.10	6.36	6.53	0.10	14.65	31.53	0.10	0.92	0.57	2.06
	30s	131,851	70,297	18,015	3090	9085	21,327	5433	7792	173	20,508	43,392	83	888	470	1970
			53.32	13.66	2.34	6.89	16.18	4.12	5.91	0.13	15.55	32.91	0.06	0.67	0.36	1.49
	40s	89,188	43,456	8906	1687	4185	12,729	2591	4738	197	13,515	28,059	43	681	250	967
			48.72	9.99	1.89	4.69	14.27	2.91	5.31	0.22	15.15	31.46	0.05	0.76	0.28	1.08
	50s	70,098	30,876	4071	621	1950	9031	981	3224	192	10,421	21,458	18	489	81	564
			44.05	5.81	0.89	2.78	12.88	1.40	4.60	0.27	14.87	30.61	0.03	0.70	0.12	0.80
	60s	55,183	18,291	1611	181	613	5373	298	1885	157	5407	13,097	5	406	33	428
			33.15	2.92	0.33	1.11	9.74	0.54	3.42	0.28	9.80	23.73	0.01	0.74	0.06	0.78
	70s	23,913	5709	395	28	112	1530	47	493	44	1343	4118	3	297	13	170
			23.87	1.65	0.12	0.47	6.40	0.20	2.06	0.18	5.62	17.22	0.01	1.24	0.05	0.71
	80s	6720	1250	103	6	16	245	6	97	26	242	864	0	125	7	52
			18.60	1.53	0.09	0.24	3.65	0.09	1.44	0.39	3.60	12.86	0.00	1.86	0.10	0.77
	Sub-total	496,718	239,123	61,695	10,736	33,402	72,002	16,958	26,035	918	68,796	148,282	272	4005	1557	6604
			48.14	12.42	2.16	6.72	14.50	3.41	5.24	0.18	13.85	29.85	0.05	0.81	0.31	1.33

* 6 STIs; *N. gonorrhoeae*, *C. trachomatis*, *M. genitalium*, *T. vaginalis*, *T. pallidum*, HSV II. Abbreviations: NG, *Neisseria gonorrhoeae*; CT, *Chlamydia trachomatis*; UU, *Ureaplasma urealyticum*; MG, *Mycoplasma genitalium*; MH, *Mycoplasma hominis*; TV, *Trichomonas vaginalis*; UP, *Ureaplasma parvum*; GV, *Gardnerella vaginalis*; TP, *Treponema pallidum*; CA, *Candida albicans*; HSVI, Herpes simplex virus type I; HSVII, Herpes simplex virus type II.

### 3.5. Co-Infection Patterns (Table 3)

Across all pathogens, *U. parvum* and *G. vaginalis* consistently represented the dominant pair in females across all age groups, accounting for 43.63% of adolescent and 42.17% in their 20s. The most frequent triplets also involved *U. parvum*, *G. vaginalis*, and either *M. hominis* or *C. albicans*, reflecting the contribution of urogenital commensals.

In males, *U. urealyticum*/*G. vaginalis* and *U. parvum*/*G. vaginalis* were comparably dominant pairs, with no significant difference across age groups. The most frequent triplet in males was *U. urealyticum*/*G. vaginalis*/*M. hominis* across all age groups.

In contrast, when restricting the analysis to the six traditional STIs (*C. trachomatis*, *N. gonorrhoeae*, *M. genitalium*, *T. vaginalis*, *T.* pallidum, and HSV II), the leading pairs differed substantially by age and sex. Among females, *C. trachomatis*/*M. genitalium* predominated in 10s (3.38%) and 20s (1.18%), whereas *C. trachomatis*/HSV II or *M. genitalium*/HSV II became more apparent in older groups. *N. gonorrhoeae* was the most common additional pathogen in triplets of the 10s. In males, *C. trachomatis*/*N. gonorrhoeae* was consistently the most frequent traditional STI pair, particularly reaching 4.25% in 10s. The most frequent triplets also involved *C. trachomatis*, *N. gonorrhoeae*, and *M. genitalium*, although their prevalence was <0.5% of all tests. Triplets involving HSV II ranked second and third, most frequently in combination with *N. gonorrhoeae*.

**Table 3:** Top Co-Infection Pairs and Triplets by Age Group and Sex.

Sex	Age	All Pathogens (Pair/Triplet)	n	%	6 STIs * (Pair/Triplet)	n	%
Female	10s	*U. parvum*/*G. vaginalis*	8223	43.63	*C. trachomatis*/*M. genitalium*	638	3.38
		*U. parvum*/*G. vaginalis*/*M. hominis*	3089	16.39	*C. trachomatis*/*M. genitalium*/*N. gonorrhoeae*	47	0.25
	20s	*U. parvum*/*G. vaginalis*	87,855	42.17	*C. trachomatis*/*M. genitalium*	2456	1.18
		*U. parvum*/*G. vaginalis*/*C. albicans*	28,490	13.68	*C. trachomatis*/*M. genitalium*/HSV II	160	0.08
	30s	*U. parvum*/*G. vaginalis*	78,657	33.96	*C. trachomatis*/*M. genitalium*	473	0.20
		*U. parvum*/*G. vaginalis*/*C. albicans*	20,052	8.66	*C. trachomatis*/*M. genitalium*/HSV II	30	0.01
	40s	*U. parvum*/*G. vaginalis*	73,937	37.69	*M. genitalium*/ HSV II	163	0.08
		*U. parvum*/*G. vaginalis*/*C. albicans*	15,856	8.08	*C. trachomatis*/*M. genitalium*/HSV II	14	0.01
	50s	*U. parvum*/*G. vaginalis*	43,939	30.24	*C. trachomatis*/HSV II	86	0.06
		*U. parvum*/*G. vaginalis*/*M. hominis*	9627	6.63	*C. trachomatis*/*M. genitalium*/*T. vaginalis*	2	0.00
	60s	*U. parvum*/*G. vaginalis*	11,261	15.60	*C. trachomatis*/HSV II	45	0.06
		*U. parvum*/*G. vaginalis*/*M. hominis*	2482	3.44	*C. trachomatis*/*M. genitalium*/HSV II	4	0.01
	70s	*U. parvum*/*G. vaginalis*	1893	8.27	*C. trachomatis*/HSV II	11	0.05
		*U. parvum*/*G. vaginalis*/*M. hominis*	346	1.51	*NA* **		
	80s	*U. parvum*/*G. vaginalis*	391	5.31	HSV II/*T. vaginalis*	2	0.03
		*U. parvum*/*G. vaginalis*/*M. hominis*	72	0.98	*NA* **		
Male	10s	*U. urealyticum*/*G. vaginalis*	543	8.24	*C. trachomatis*/*N. gonorrhoeae*	280	4.25
		*U. urealyticum*/*G. vaginalis*/*M. hominis*	169	2.56	*C. trachomatis*/*N. gonorrhoeae*/*M. genitalium*	33	0.50
	20s	*U. urealyticum*/*G. vaginalis*	11,857	10.48	*C. trachomatis*/*M. genitalium*	1536	1.36
		*U. urealyticum*/*G. vaginalis*/*M. hominis*	3654	3.23	*C. trachomatis*/*N. gonorrhoeae*/*M. genitalium*	160	0.14
	30s	*U. parvum*/*G. vaginalis*	13,558	10.28	*C. trachomatis*/*N. gonorrhoeae*	787	0.60
		*U. urealyticum*/*G. vaginalis*/*M. hominis*	3890	2.95	*C. trachomatis*/*N. gonorrhoeae*/*M. genitalium*	60	0.05
	40s	*U. parvum*/*G. vaginalis*	9067	10.17	*C. trachomatis*/*N. gonorrhoeae*	386	0.43
		*U. urealyticum*/*G. vaginalis*/*M. hominis*	2363	2.65	*C. trachomatis*/*N. gonorrhoeae*/*M. genitalium*	27	0.03
	50s	*U. parvum*/*G. vaginalis*	7117	10.15	*C. trachomatis*/*N. gonorrhoeae*	124	0.18
		*U. urealyticum*/*G. vaginalis*/*M. hominis*	1603	2.29	*C. trachomatis*/*N. gonorrhoeae*/*M. genitalium*	8	0.01
	60s	*U. parvum*/*G. vaginalis*	3557	6.45	*C. trachomatis*/*N. gonorrhoeae*	32	0.06
		*U. urealyticum*/*G. vaginalis*/*M. hominis*	898	1.63	*C. trachomatis*/*N. gonorrhoeae*/*M. genitalium*	3	0.01
	70s	*U. urealyticum*/*G. vaginalis*	923	3.86	*C. trachomatis*/*N. gonorrhoeae*	4	0.02
		*U. urealyticum*/*G. vaginalis*/*M. hominis*	218	0.91	*NA* **		
	80s	*U. parvum*/*G. vaginalis*	151	2.25	*C. trachomatis*/*M. genitalium*	2	0.03
		*U. urealyticum*/*G. vaginalis*/*M. hominis*	46	0.68	*NA* **		

* 6 STIs; *N. gonorrhoeae*, *C. trachomatis*, *M. genitalium*, *T. vaginalis*, *T. pallidum*, HSV II. ** NA, not applicable.

### 3.6. Inter-Pathogen Correlation Characteristics (Table 4)

In females, the strongest correlations were observed among flora-associated organisms, such as *U. parvum*/*G. vaginalis* (*φ* = 0.24–0.35) and *U. urealyticum*/*M. hominis* (*φ* = 0.22–0.30), consistently across age groups. In males, correlations were similarly dominated by flora-associated organisms, particularly *U. parvum*/*G. vaginalis* (*φ* = 0.29–0.34), *M. hominis*/*G. vaginalis* (*φ* = 0.29), and *U. urealyticum*/*M. hominis* (*φ* = 0.28–0.33) also observed across age groups.

The strongest correlations were *U. parvum*/*G. vaginalis* in females aged 60s (*φ* = 0.35) and in males aged 50s (*φ* = 0.34), further underscoring the predominance of commensal organism associations. *C. trachomatis*/*M. hominis* and *C. trachomatis*/*U. urealyticum* were noted in female and male adolescents, respectively, with moderate correlation strength (*φ* = 0.28 and 0.25). No consistent negative correlations (*φ* ≤ −0.05) were observed.

Taken together, these findings indicate that co-infection networks are primarily driven by commensal organisms, while clinically relevant correlations between STIs, especially involving *C. trachomatis*, occur mainly in younger individuals.

**Table 4:** Top inter-pathogen correlations (φ ≥ 0.20) by age group and sex.

Age	Female	*φ*	Male	*φ*
10s	*U. urealyticum*/*M. hominis*	0.30	*M. hominis*/*G. vaginalis*	0.29
	*C. trachomatis*/*M. hominis*	0.28	*C. trachomatis*/*U. urealyticum*	0.25
20s	*U. urealyticum*/*M. hominis*	0.24	*U. parvum*/*G. vaginalis*	0.30
	*U. parvum*/*G. vaginalis*	0.24	*M. hominis*/*G. vaginalis*	0.29
30s	*U. parvum*/*G. vaginalis*	0.25	*U. parvum*/*G. vaginalis*	0.30
	*U. urealyticum*/*M. hominis*	0.23	*U. urealyticum*/*M. hominis*	0.28
40s	*U. parvum*/*G. vaginalis*	0.28	*U. parvum*/*G. vaginalis*	0.32
	*U. urealyticum*/*M. hominis*	0.25	*U. urealyticum*/*M. hominis*	0.29
50s	*U. parvum*/*G. vaginalis*	0.34	*U. parvum*/*G. vaginalis*	0.34
	*U. urealyticum*/*M. hominis*	0.25	*U. urealyticum*/*G. vaginalis*	0.30
60s	*U. parvum*/*G. vaginalis*	0.35	*U. parvum*/*G. vaginalis*	0.33
	*U. urealyticum*/*G. vaginalis*	0.25	*U. urealyticum*/*G. vaginalis*	0.30
70s	*U. parvum*/*G. vaginalis*	0.31	*U. urealyticum*/*G. vaginalis*	0.30
	*U. urealyticum*/*M. hominis*	0.22	*U. urealyticum*/*M. hominis*	0.29
80s	*U. parvum*/*G. vaginalis*	0.26	*U. urealyticum*/*M. hominis*	0.33
	*M. hominis*/*U. parvum*	0.22	*U. parvum*/*G. vaginalis*	0.29

### 3.7. Age-Stratified Co-Infection Coverage (Supplementary Table S1)

Coverage was calculated as the sum of the top five co-infection pairs (or triplets) divided by the total number of tests in each age group ×100. In females, cumulative coverage of the top five pairs and triplets reached 135.92% and 67.89% in adolescents, and 113.24% and 45.65% in the 20s, whereas in males, the corresponding values were 32.47% and 9.67% in adolescents and 32.39% and 7.94% in the 20s. Coverage of both pairs and triplets declined progressively with age, falling to 14.48% and 2.87% in the 80s in females, and to 6.73% and 1.31% in the 80s in males, indicating frequent multiple co-detections in younger age groups.

Co-infections among the six traditional STIs (*C. trachomatis*, *N. gonorrhoeae*, *M. genitalium*, *T. vaginalis*, *T. pallidum*, and HSV II) were markedly lower. Males consistently showed higher coverage rates than females, ranging from 8.09% in adolescents to 0.35% in individuals in their 50s, despite a clear age-related decline in both sexes. These findings suggest that traditional STI co-infections are largely confined to adolescents and young adults, occur more frequently in males, and contrast with the broader, commensal-driven co-infection patterns observed across all age groups.

### 3.8. Hub Pathogen Dynamics (Supplementary Table S2)

Across all age groups, *G. vaginalis* appeared in four to five of the top five pairs, underscoring its role as the central hub organism of co-infection. *U. parvum* was the second most consistent partner, particularly dominant in reproductive-aged women (20–40s). In contrast, *C. albicans* showed a declining presence in older age groups, while HSV II emerged as a hub pathogen from the 60s in females and the 50s in males, reflecting a shift in co-infection diversity with advancing age. Notably, *M. hominis* ranked as the third most consistent hub organism, being represented across all age groups and sustaining its presence into older adulthood, where it became relatively more prominent, together with HSV II.

## 4. Discussion

This nationwide four-year analysis of approximately 1.4 million multiplex PCR tests provides novel insights into the epidemiology and co-infection dynamics of 12 urogenital and STI pathogens in Korea. To our knowledge, this is the first study to leverage such a large-scale, longitudinal dataset to investigate multiplex PCR-based urogenital and STIs surveillance, enabling population-level patterns and co-infection trends that have not been previously reported. Our findings underscore several key epidemiologic patterns with important clinical and public health implications. Urogenital flora-associated organisms and traditional STIs should be comprehensively analyzed both collectively and separately, given their distinct clinical and epidemiological significance across different sexes and age groups.

The overall positivity rate for at least one pathogen among the 12 tested urogenital and STI pathogens was notably high (67.23%), while traditional STIs alone accounted for a much lower rate (10.54%).

### 4.1. Temporal Trends and Stability

Despite increasing test volumes between 2021 and 2024, positivity for most pathogens declined significantly, suggesting improvements in prevention, diagnosis, or treatment during this period [1,3].

However, *C. albicans* positivity increased in women (*p* = 0.0058), which may reflect shifts in gynecologic infection dynamics, including rising rates of vulvovaginal candidiasis (VVC) and recurrent VVC [4,5], contrasting with the age-stratified analysis that showed a decline with advancing age and suggesting shifting risk exposures in younger reproductive-age groups. Given that *C. albicans* accounts for more than 75% of VVC cases—with the remainder caused by non-*albicans Candida* species—and affects up to 9% of women with recurrent episodes, this trend may signal growing challenges in antifungal resistance, hormonal influences, and hygiene-related factors. Importantly, recent global studies have reported a higher risk of genital *Candida* infections among individuals with type 2 diabetes, particularly those receiving sodium–glucose cotransporter-2 (SGLT2) inhibitor therapy, owing to glucosuria, altered mucosal immunity, and enhanced fungal proliferation under hyperglycemic conditions [6,7,8,9,10,11,12]. Although such associations have not yet been systematically investigated in Korean populations, the national prevalence of diabetes continues to rise—affecting approximately 15% of adults aged ≥ 30 years during 2021–2022 [3]—and the prescription of SGLT2 inhibitors has expanded rapidly in recent years, increasing nearly nine-fold between 2015 and 2020 (1.2% → 10.5%) [13,14]. These trends may collectively contribute to the increasing detection of *C. albicans* in urogenital specimens. Further investigation into diabetes- and SGLT2-related susceptibility, antifungal resistance, and host–pathogen interactions is warranted, particularly in reproductive-age women. The clinical burden includes significant discomfort, reduced quality of life, and increased healthcare costs, underscoring the need for improved diagnostic and therapeutic strategies [15].

Conversely, HSV II positivity declined in men, aligning with prior reports of decreasing seroprevalence. This trend may reflect enhanced public health interventions, increased condom use, and greater awareness of genital herpes transmission. However, HSV II remains clinically significant due to lifelong latency, frequent asymptomatic shedding, and associations with male infertility and HIV acquisition risk [16,17]. The observed decline should be interpreted cautiously, as subclinical infections and underdiagnosis remain common in both serologic surveillance and molecular diagnostics.

Monthly and seasonal analyses revealed no significant variation, indicating that the burden of urogenital and STIs in Korea remains relatively stable year-round. This suggests that behavioral rather than environmental factors are primary drivers of transmission [3]. Moreover, it is important to consider the diversity of transmission routes—including vaginal, anal, and oral intercourse, as well as vertical transmission during childbirth. These varied pathways may influence pathogen distribution and detection patterns, particularly for organisms such as HSV I and *C. albicans*, which are often under-recognized in traditional STI panels.

While our findings contrast with studies reporting seasonal peaks in STIs such as syphilis and gonorrhea—particularly during warmer months in China and other regions [18,19]—the absence of seasonality may reflect consistent sexual behavior patterns, stable healthcare access, or limitations in temporal resolution. Further research is warranted to explore subpopulation-specific or pathogen-specific seasonal trends that may be masked in aggregate-level analyses.

### 4.2. Sex- and Age-Specific Differences

Pronounced sex-based differences were evident: females showed significantly higher positivity for the broader urogenital and STI panel (77.73%) compared to males (48.14%), whereas detection rates for traditional STIs were higher in males (9.51% in females vs. 12.42% in males).

These findings suggest that urogenital commensals such as *G. vaginalis* and *U. parvum* may be more prevalent in females, reflecting non-traditional STI dynamics, potentially due to anatomical, behavioral, or microbiome-related factors. In contrast, males exhibited higher positivity for traditional bacterial STIs such as *C. trachomatis* and *N. gonorrhoeae*, consistent with prior epidemiologic patterns observed across diverse populations and highlights the limitations of relying solely on traditional STI panels [2,3].

Although annual trends indicated a general decline in positivity for most pathogens, these sex-specific differences persisted across the study period. This suggests that population-level improvements in prevention and care may mask ongoing disparities between men and women. Expanding diagnostic coverage to include commensal and emerging pathogens is particularly critical for female populations, where the burden of infection may be underestimated. Collectively, these results underscore the need for sex- and age-tailored diagnostic strategies and public health interventions [20,21,22].

### 4.3. Age-Stratified Analysis

Age-stratified analysis confirmed that adolescents and young adults are disproportionately affected by traditional STIs such as *C. trachomatis* and *N. gonorrhoeae* [1,2,22,23], whereas commensals such as *G. vaginalis* and *U. parvum* were consistently detected across all reproductive age groups [1,2,3].

This pattern complements the temporal analysis, in which overall positivity declined annually, but age-specific peaks in adolescents and young adults remained pronounced, highlighting the coexistence of broad declines with subgroup-specific vulnerabilities. Such a distribution likely explained by a combination of biological susceptibility (e.g., cervical ectopy in adolescent females) and behavioral risk factors such as low condom use and limited STI awareness [23,24]. Restricted access to screening services further exacerbates vulnerability in younger populations.

These findings highlight the importance of age-targeted screening and youth-friendly sexual health services to improve early detection and reduce transmission.

### 4.4. Co-Infection Dynamics

Analysis of co-infections provides clinically relevant insights into pathogen interactions and diagnostic complexity. Although traditional STI–STI co-infections were relatively rare, combinations such as *C. trachomatis*/*M. genitalium* in females and *C. trachomatis*/*N. gonorrhoeae* in males were disproportionately concentrated in adolescents and young adults [1,2]. These findings support targeted screening in younger populations, where co-infections may exacerbate disease severity, complicate treatment, and increase transmission risk [21]. Co-infections can amplify mucosal inflammation, facilitate HIV acquisition, and contribute to diagnostic delays due to overlapping or atypical symptoms [25,26].

Across all pathogens, *U. parvum*/*G. vaginalis* was the dominant pair in females, while *U. urealyticum*/*G. vaginalis* and *U. parvum*/*G. vaginalis* were comparably frequent in males. Triplets involving commensals (*U. parvum*, *G. vaginalis*, *M. hominis*, or *C. albicans*) were also common in females. These patterns suggest that most co-detections are driven by flora-associated organisms rather than traditional STIs [5,14].

Although often asymptomatic, commensals can contribute to dysbiosis, recurrent symptoms, and diagnostic ambiguity—especially when detected via multiplex PCR without clear clinical correlation [27]. In reproductive-age individuals, frequent co-detection of *U. parvum* and *G. vaginalis* may have implications for maternal health, as these organisms have been associated with subclinical inflammation, cervical epithelial disruption, and adverse pregnancy outcomes such as preterm birth and premature rupture of membranes, consistent with prior evidence linking commensal colonization to reproductive complications [28,29,30].

These findings highlight the need for targeted screening in pregnant populations and for careful interpretation of test results, integration of clinical context, and development of refined diagnostic algorithms to distinguish colonization from true infection.

### 4.5. Coverage and Hub Dynamics

Coverage of the top five pathogen pairs was highest among younger individuals of both sexes, with males consistently showing higher rates for traditional STIs across all age groups. Notably, coverage declined sharply with age, falling below 20% in individuals over 80, highlighting that multi-pathogen colonization and infection are concentrated in younger populations.

The consistently higher coverage of traditional STIs in males across age groups may reflect behavioral and anatomical factors, such as higher rates of unprotected sexual activity, lower healthcare-seeking behavior, and the absence of routine screening programs targeting asymptomatic infections in men. Additionally, the male urogenital tract may provide a distinct ecological niche that facilitates persistent colonization by certain pathogens, contributing to elevated co-infection rates. These findings underscore the need for targeted public health interventions and gender-specific surveillance strategies [30,31,32].

Hub analysis identified *G. vaginalis* as the central node across all age groups, consistent with its reported role in bacterial vaginosis and vaginal dysbiosis [33]. Its persistent presence and frequent co-detection suggest that *G. vaginalis* may act as a microbial anchor in polymicrobial communities, facilitating biofilm formation and modulating the local immune environment [34]. However, this apparent centrality likely reflects its high baseline prevalence and colonization nature rather than causal dominance. Therefore, the hub status of *G. vaginalis* should be interpreted with caution, as it may represent ecological co-occurrence rather than direct pathogenic interaction, and further mechanistic studies are warranted to clarify these relationships.

In reproductive-age individuals, *U. parvum* emerged as a frequent partner node, particularly in females. Though often considered a commensal, *U. parvum* has been implicated in subclinical inflammation, cervical epithelial disruption, and adverse pregnancy outcomes such as preterm birth and premature rupture of membranes [35,36]. Its consistent co-detection with *G. vaginalis* may reflect synergistic colonization patterns that complicate diagnosis and management.

In older adults, *M. hominis* and HSV II gained prominence in both sexes, indicating a shift toward more diverse and potentially pathogenic co-infections with age [37,38]. Age-related immune senescence may contribute to HSV II reactivation and atypical presentations, while *M. hominis* has been associated with infertility, spontaneous abortion, and systemic complications in immunocompromised hosts [5]. These findings highlight the evolving nature of genital tract microbial networks across the lifespan and emphasize the need for age-tailored diagnostic interpretation and clinical vigilance.

### 4.6. Correlation Characteristics

The observed correlation patterns closely align with age-stratified prevalence and co-infection trends. Strong associations among commensal organisms—particularly *U. parvum*/*G. vaginalis* and *U. urealyticum*/*M. hominis*—reflect flora-driven colonization in reproductive-age individuals, consistent with high prevalence and frequent triplet co-detections.

In contrast, traditional STI–STI associations were limited but clinically relevant in younger populations. Moderate correlations involving *C. trachomatis* in adolescents mirror the concentrated burden of traditional STIs in this age group, where behavioral risk factors and mucosal susceptibility converge.

Previous studies have reported rare negative associations between *C. albicans* and *N. gonorrhoeae* or *T. vaginalis*, suggesting competitive microbial interactions [5]. However, consistent negative correlations were not observed in our dataset, and further studies are warranted.

### 4.7. Strengths and Limitations

The main strength of this study lies in its unprecedented sample size and the use of a standardized multiplex PCR platform that simultaneously targeted 12 urogenital and STI pathogens across four years. This allowed detailed stratification with significant findings by sex, age, and pathogen type, providing valuable epidemiologic insights.

Nevertheless, several limitations should be acknowledged. First, the dataset was derived from laboratory records without accompanying clinical information—such as patients’ symptoms, underlying comorbidities (e.g., diabetes, pregnancy status, immunocompromise), or treatment history—which limits interpretation of clinical relevance, including disease severity, therapeutic outcomes, and correlations between laboratory detection and symptomatic infection. Second, repeated tests from the same individual could not be identified, precluding longitudinal follow-up of reinfection or treatment response. Finally, the cross-sectional design precludes inference of causality or population-level transmission dynamics.

Furthermore, diagnostic sensitivity may vary according to specimen type. Although urine is widely used for molecular detection of *C. trachomatis* and *N. gonorrhoeae* in men, previous studies have shown that penile-meatal or urethral swabs may yield slightly higher sensitivities than urine for several urogenital pathogens, including *C. trachomatis*, *N. gonorrhoeae*, and *T. vaginalis* [39,40]. In addition, *T. pallidum* detection by PCR in this study should be interpreted with caution, as urine-based assays have limited sensitivity compared with lesion-derived specimens, and thus the diagnostic yield may be underestimated, noting that the clinical prevalence of syphilis in Korea is very low (latent syphilis reported at ~5.29 per 100,000 in 2021) [41]. Therefore, some of the lower positivity observed in males may partly reflect sample-type–related detection differences rather than true epidemiologic disparities.

### 4.8. Conclusions and Implications

In summary, this nationwide analysis revealed distinct sex- and age-specific distributions of urogenital and STI pathogens in Korea, a declining prevalence of traditional STIs, and commensal-driven co-infection networks. Adolescents, young adults, and males were identified as the highest-risk groups for both traditional STIs and multi-pathogen co-detections. Notably, *C. albicans* positivity increased over time in women despite age-related declines, and *G. vaginalis* consistently emerged as a central hub organism, underscoring the evolving complexity of urogenital microbial ecology. These findings highlight the need for targeted screening in younger populations and women of reproductive age, and emphasize the value of multiplex PCR as a surveillance tool to capture the complexity of co-infection networks. Future studies integrating clinical and behavioral data with laboratory findings will be critical to fully understand the drivers of the urogenital and STI transmission and to optimize prevention strategies.

## Data Availability

The original contributions presented in this study are included in the article/Supplementary Materials. Further inquiries can be directed to the corresponding author.

## Supplementary Materials

The following supporting information can be downloaded at: https://www.mdpi.com/article/10.3390/pathogens14111073/s1, Table S1: Coverage of Top 5 Co-Infection Pairs and Triplets; Table S2: Age-Specific Dynamics of Hub Pathogens; Table S3; Detailed *p*-values for statistical comparisons of positivity rates by sex, year, and age group.
